# The use of 5-azacytidine in pregnant patient with Acute Myeloid Leukemia (AML): a case report

**DOI:** 10.1186/s12884-019-2522-1

**Published:** 2019-10-31

**Authors:** Abdullah M. Alrajhi, Sarah A. Alhazzani, Nouf M. Alajaji, Fouad H. Alnajjar, Nawal F. Alshehry, Imran K. Tailor

**Affiliations:** 1 Department of Pharmacy, King Fahad Medical City, Riyadh, Saudi Arabia; 2 Maternal Fetal Medicine Department, King Fahad Medical City, Riyadh, Saudi Arabia; 3 Comprehensive Cancer Center, King Fahad Medical City, Riyadh, Saudi Arabia

**Keywords:** “5-azacyitidne, Acute Myeloid leukemia, pregnancy, third trimester, Daunorubicin, Cytarabine, Leukemia”

## Abstract

**Background:**

The management of Acute Myeloid Leukemia (AML) during pregnancy remains challenging as both the maternal and fetal outcomes should be considered. Several reports suggested that chemotherapy can be administered safely during the second and third trimester of pregnancy. However, the use of 5-azacytidine presents limitation due to lack of data.

**Case presentation:**

A 28-years-old woman in the 26th week of gestation diagnosed with FLT3/ITD-mutated AML, complete remission was induced by Daunorubicin and Cytarabine, and subsequently with 5-azacytidine (75 mg/m2 daily for 7 days) with no fetal hematological or toxicity issues. Fetal ultrasound showed no aberrant morphology. Fetal size below the 5th percentile with normal umbilical artery dopplers, normal middle cerebral artery dopplers and ductus venosus doppler.

Three weeks post 5-azacytidine, the team determined the most appropriate time for delivery after balancing the risks of prematurity and prevention of disease relapse since patient in hematological remission. The patient underwent elective lower segment caesarian section and had a baby girl delivered at 35 weeks of gestation weighing 1670 g without apparent anomalies.

**Conclusion:**

Treatment using 5-azacytadine during last trimester of pregnancy resulted in no major fetal and maternal complications. These findings concluded that 5-azacytadine during the third trimester of pregnancy seems to be safe however, potential risks of this agent should be considered.

## Background

AML accounts for two-thirds of all acute leukemia cases reported during pregnancy [1–3]. The incidence of leukemia during pregnancy is reported to be 1 in 75,000–100,000 pregnancies. However, data regarding the management of acute myeloid leukemia (AML) during pregnancy is limited, mostly to case reports and retrospective studies, which poses difficulties in establishing parameters for optimal therapy, maternal outcome, and fetal complications [4]. The management of AML during pregnancy is complicated and remains challenging as both the maternal and fetal outcomes should be considered [1]. Administration of chemotherapy during the first trimester of pregnancy is contraindicated due to the potential risk of teratogenicity [5]. Mothers treated in the first trimester are more likely to experience congenital malformations, spontaneous abortion, and fetal loss. However, chemotherapy can be administered safely during the second or third trimester [6, 7]. Aviles et al. described a report of 84 children who received chemotherapy in utero including 38 during the first trimester. The children were examined for growth, physical health, development, cytogenetic, hematological, neurological, psychological, and learning disorders. All of the children observed had a normal birth weight, a normal learning and educational performance, and no congenital, neurological, or psychological abnormalities were detected [8]. Long-term follow up of children exposed to chemotherapy after the first trimester does not show an increased incidence of congenital malformation, preterm delivery, growth restriction, and mental disorder as compared to nonmalignant pregnant [6, 9]. Delaying the induction of chemotherapy treatment can harmfully affect fetal growth [10]. Untreated maternal cancer can lead to adverse pregnancy outcomes, including intrauterine growth restriction (IUGR) and fetal loss [11].

There is a robust evidence regarding the safety of many chemotherapeutic agents during pregnancy. However, the use of 5-azacytidine presents limitation due to lack of data.

The purpose of this paper is to describe the treatment of AML during pregnancy with chemotherapy, including the rarely used hypomethylating agent 5-azacytidine.

## Case presentation

A 28 years old lady in her 2nd pregnancy presented to the emergency department in her 26th week of gestation with dizziness and palpitation for 2 weeks. She had no prior illness and her last pregnancy was complicated by severe preeclampsia and she was delivered by caesarian section. She was found to have severe anemia with a hemoglobin of 7.2 g/dl (11–16), WBC 57.74 × 109/l (3.9–11), platelets 362 × 109/l (155–435) with 73% circulating blasts on blood film. Renal function, liver function, echocardiogram (ECHO), and electrocardiogram (ECG) were normal. Flow cytometry on bone marrow biopsy showed 42% of total leukocytes expressed CD13, CD33, CD117, CD38, HLA-DR, MPO, CD11C partial, & CD15 partial (27%). Morphologically, the marrow showed 60% blasts thus confirming AML.

Cytogenetics and molecular tests confirmed the presence of Nucleophospmin 1 (NPM1) and FMS like Tyrosine Kinase 3 with Internal Tandem Duplication 3 (FLT3 ITD) (ratio of 0.22) mutation. Her fetal ultrasound has shown normal morphology, placentation and amniotic fluid but the fetal size was below the 5th percentile with normal umbilical artery dopplers, normal middle cerebral artery dopplers and ductus venosus doppler. Uterine artery dopplers were not done. She did not have prior ultrasound for comparison but was sure about her dates with regular periods. Thus, daily cardiotocography (CTG) and fetal ultrasound every 2–3 weeks to ensure proper growth were planned during her hospitalization.

Patient showed no signs of preeclampsia and was normotensive at 110/70 mmHg and urine was negative for protein. She received induction chemotherapy with daunorubicin and cytarabine and achieved morphological remission on day 28 marrow (residual blasts 4%). However, her induction was complicated by myopericarditis likely picture due to anthracyclines. She had chest pain, ST-T changes on ECG and ECHO was showing drop in the ejection fraction (EF) from 60 to 35% with regional wall motion abnormalities. Myopericarditis was managed with analgesia, beta blockers, steroids, and aspirin (aspirin was stopped later due to low platelets correlated to chemotherapy). She had an episode of febrile neutropenia during induction required admission to critical care unit, and was managed with appropriate antibiotics as per local protocol. She was frequently monitored by the maternal and fetal medicine team with fetal ultrasound every 2 weeks and CTG once daily. The fetus showed normal growth and wellbeing between scans. Post induction, she was given 5-azacytidine 75 mg/m2 for 7 days in order to reduce morbidity due to further intensive chemotherapy such as high dose Cytarabine and secondly as a bridge to delivery. This drug was deemed by the team to be the safest option at this stage. She was given 5-azacytidine with no hematological or toxicity issues.

Three weeks post 5-azacytidine, decision was made to deliver the baby on 35 weeks of gestation to prevent disease relapse since patient was on hematological remission. She was given dexamethasone 6 mg Intramuscularly every 12 h for 4 doses for lung maturity 1 week before delivery, then taken for elective lower segment caesarian section after counseling about the mode of delivery and she opted for elective cesarean section. The operation was uncomplicated and she delivered baby girl at 35 weeks of gestation weighing 1670 g with apgars 9 and 9 at 1 and 5 min and arterial cord PH 7.32. Hemoglobin level was 19 g/dL The baby was taken to the NICU and stayed 2 weeks due to small weight for observation. ECHO was done for the baby and showed tiny PDA left to right shunt, normal left atrium and ventricle with EF of 68% and Fractional shortening (FS) of 34%. Scheduled follow up for development was planned after 6 and 12 months. Once the patient recovered, she was given high dose cytarabine consolidation of 3 g/m2 (planned for 3 cycles of high dose cytarabine with midostaurin).

## Discussion and conclusions

We report a case of AML during pregnancy to highlight the effect of the chemotherapy, including the rarely used hypomethylating agent 5-azacytidine on the maternal and fetal outcomes. Several studies suggested that chemotherapy can be administered safely during the second and third trimester of pregnancy [6, 7]. Intrauterine growth restriction (IUGR) remains a potentially fatal complication of chemotherapy during pregnancy [12]. Cardonick and Iacobucci presented the results of 376 fetuses exposed to chemotherapy in utero and reported the following complications: IUGR (7%), preterm labor or spontaneous rupture of membranes (5%), fetal death (5%) [12]. However, Fetal growth restriction observed, in this case, was prior to administrating chemotherapy. The effect of hypomethylation agents on genetic expression and fetal development, up to our knowledge, are limited [13]. However, this concern was disclosed with patient before proceeding with the treatment.

To our knowledge, this is the second reported case of a pregnant woman being successfully treated with 5-azacytidine with no major fetal and maternal complications.

The first case report of 5-azacytidine treatment during pregnancy was reported by Mahdi, et al. (2016) [14]. A 39-years-old woman in the 30th week of her first pregnancy diagnosed with FLT3/ITD-mutated AML, was orally administrated sorafenib 400 mg twice daily and subcutaneously administered 5-azacytidine 75 mg/m^2^/day for 7 days. The patient was successfully treated with no adverse fetal outcomes that have been noted after early follow-up and a healthy newborn was delivered.

The standard regimen for remission-induction consists of a combination of anthracycline and Cytarabine [15]. This combination can be administered safely in pregnancy especially during the second or third trimesters [16]. Some anthracyclines, such as doxorubicin and daunorubicin are safer during pregnancy. Idarubicin is more lipophilic and has an increased risk of crossing the placenta with a higher rate of potential fetal complications than the other anthracyclines [17, 18]. Therefore, idarubicin should be avoided during pregnancy.

Human data regarding the safety of 5-azacytidine during pregnancy is lacking but the safety and toxicity of 5-azacytidine during pregnancy have been studies in both mice and rats with a dose-dependent decrease in fetal survival/ body weight, and a high incidence of microphthalmia and exencephaly [19, 20]. 5-azacytidine was also associated with neuronal cell apoptosis in mice during the late weeks of pregnancy [21]. Data derived from animal models is, however, hard to be extrapolated to the human pregnancy; and the doses used in humans are usually far lower than those known to be teratogenic in animals [22].

In conclusion, there is insufficient data to guide practice regarding optimal therapy of AML during pregnancy. We are describing the safe treatment of AML with 5-azacytidine and in late stages of pregnancy in our case and a previously reported similar case [7]. The need for more evidence to evaluate the general safety of 5-azacytidine in early pregnancy is of paramount importance.

## Data Availability

The datasets used and/or analyzed during the current case report are available from the corresponding author on reasonable request.

## Abbreviations

AML: Acute myeloid leukemia
CTG: Cardiotocography
ECG: Electrocardiogram
ECHO: Echocardiogram
FS: Fractional shortening
IUGR: Intrauterine growth restriction
NPM1: Nucleophospmin 1
